# Improving Work Ability of Mentally Burdened Emerging Adults in Vocational Integration Programs: A Study Protocol

**DOI:** 10.3389/fpsyg.2019.01391

**Published:** 2019-06-19

**Authors:** Filomena Sabatella, Agnes von Wyl

**Affiliations:** Psychological Institute, Zurich University of Applied Sciences, Zurich, Switzerland

**Keywords:** mental health, emerging adults, vocational integration program, unemployment, psychotherapy, feasibility study

## Abstract

**Background:** Emerging adults who do not successfully transition from compulsory education to the upper secondary level have a greater risk of developing mental illness than their working peers. Early intervention is important in these cases; the course of an illness can be positively influenced if treated early and without delay, and consequently, the illness will not affect the professional and personal development of the emerging adults any further. Postponing treatment of a mental illness increases the risk of chronification of the disease. Up to now, vocational integration programs have been mostly educationally oriented. To recognize and treat mental illness in a timely way, we developed an intervention that includes psychotherapy support in a vocational integration program for emerging adults who have not successfully transitioned from compulsory education to the upper secondary level.

**Aim:** The aim of this study is to investigate the effectiveness of the described intervention, which will be delivered to all emerging adults aged 16–29 entering the vocational integration program. Another objective is to determine if it is worthwhile and feasible to proceed with testing the intervention in a full-scale clinical trial. Conducting research with this population can be very difficult, which makes it especially important to test the feasibility of a clinical trial.

**Methods:** A single group clinical trial will be conducted to examine the effectiveness of the intervention described above. Emerging adults aged 16–29 entering a vocational integration program will be recruited. Participants will provide informed consent and complete pre-intervention measures; they then take part in the intervention group in addition to the regular vocational integration program. The primary outcomes for this study are an increase in work ability, mental health literacy, and mental health. Demographic information, vocational biography, and satisfaction with the intervention will also be collected. Questionnaire data will be collected at pre-intervention, post-intervention, and 6-month follow-up.

**Ethics and dissemination:** The study was approved by the Ethics Committee of the Canton of Zurich (2017-00936) and registered in https://clinicaltrials.gov (NCT03328286) before recruitment started. The results will be reported in future scientific publications and international conference presentations.

## Introduction

In Switzerland, after 9 years of compulsory education, 53% of young adults start an apprenticeship, 31% enroll in an upper secondary school, and 16% do not have an interim solution (Keller, 2014). This means that in Switzerland, every sixth young adult does not transition directly from compulsory education to upper secondary education/training. For young people who, after completing lower secondary education, cannot immediately commence vocational education and training (VET) or enroll in a school offering general upper secondary education, various transitional options for vocational integration are available.

Vocational integration programs are voluntary interim solutions for young people who for various reasons do not find an apprenticeship or secondary school after completing compulsory schooling. The programs have been designed to facilitate the transition to vocational or school-based training (Annen et al., 2010). The training period is usually limited to 1 year. The focus of the various programs can vary: there are more compensatory, remedial programs that focus on school, language, or other deficits. Some young people use the year in the bridge year course as a decision-making aid when choosing a career. For others, it is a buffer to bridge the waiting period until the start of their apprenticeship or secondary school. Overall, the programs all have the same aim: vocational integration (Meyer, 2004).

A look at the mental health of the participants of these programs reveals a significantly higher percentage of mental distress among the emerging adults than among their peers in apprenticeships or at upper secondary schools (Sabatella and von Wyl, 2014): the study found that 74% of the participants showed signs of mental distress. The causes of this higher percentage are twofold: for one, not finding training/workplace is a burden. For another, emerging adults who already have mental distress may struggle with finding an apprenticeship (Schaufeli, 1997).

Regardless of the cause, the consequences are the same: for unemployed emerging adults with severe mental health problems, it is difficult to find an apprenticeship (OECD, 2014). Many of these emerging adults have a mental disorder that has not been diagnosed or treated. This fact is reflected in the increasing number of emerging adults receiving a disability pension due to mental health problems. Although the total number of disability pensions has decreased since 2005 due to several reforms, there is no decrease among the youngest age group. Since 1995, newly awarded pensions due to mental disorders among the youngest disability pension recipients (aged 18–19) have registered a yearly increase of 6%; among 20- to 24-year-olds, the yearly increase is 2% (OECD, 2014). This is made worse by the fact that emerging adults often do not seek professional help when they experience mental health problems. The reasons for this can be manifold: negative beliefs and attitude toward professional help-seeking, fear of the stigma of mental health problems, a lack of emotional competence, or help-negation when help is needed the most (Rickwood et al., 2005). Baer et al. (2016) analyzed 400 dossiers of emerging adults who had been awarded a disability pension between 2010 and 2013 due to mental health problems and found that the pensions had been awarded before their 23rd birthday, and only 14% had completed an apprenticeship. The researchers suggested different measures: prioritization of completing an apprenticeship, systematic interdisciplinary assessment, and early recognition and intervention of mental disorders in schools and vocational training.

In vocational integration programs, emerging adults work on skills needed for entering the labor market. In the program in which we are conducting our study, participants learn how to write an application dossier and how to conduct a job interview, but they also work on educational deficits, such as in general knowledge and mathematics. In addition, they have regular external work assignments so that they get to know the job for which they are applying. To strengthen the emerging adults in vocational integration programs, the intervention in this study targets other aspects, such as work ability, mental health literacy, and mental health. We assume that the psychotherapeutic services that we plan to provide will lead to improvement in the participants’ work ability, mental health literacy, and mental health and therefore, together with the support of the vocational integration program, will facilitate the emerging adults’ integration in the labor market.

Work ability is defined as the ability to master certain tasks in very specific situations (Prümper and Richenhagen, 2011), the skill to master tasks in a certain moment in time (Ilmarinen et al., 2005). Historically, the term work ability is strongly connected with the Work Ability Index (Tuomi et al., 1994), and it is often operationalized using that measure.

To assess the participants’ work ability more thoroughly, we will also measure their self-esteem and self-efficacy. Self-esteem and self-confidence have a central place in psychology in general and in relation to almost all disorders in particular (Potreck-Rose and Jacob, 2015). Self-esteem is seen as a psychosocial resource. A lack of this resource is often related to various mental disorders, such as depression (Turner et al., 2004) or eating disorders (Brockmeyer et al., 2013).

Self-efficacy is similarly relevant. In view of the fact that self-esteem and experiencing self-efficacy are essential dimensions of the ability to work and that self-efficacy plays a significant role, especially for (re-)entrants into everyday working life (e.g., after a period of illness) (Black et al., 2018), it is worth taking a closer look at these dimensions. “Self-efficacy,” coined by Bandura in 1977, plays a major role in Bandura’s social cognitive theory (Bandura, 2001). Bandura found that heightened perceived self-efficacy before the start of psychotherapy and an increase in perceived self-efficacy during the therapeutic intervention were important predictors of the success of the treatment. A newer study has confirmed that for emerging adults with a diagnosed mental disorder, work is a resource. It gives them the possibility of becoming financially independent, and they experience an increase in their self-esteem and self-efficacy (Torres Stone et al., 2016). Early sensitization or early intervention services for mental disorders are generally recommended (Chan et al., 2015); however, such psychotherapeutic services focus less on self-esteem and self-efficacy and more on health competencies (knowledge in the field of mental health, help-seeking behavior, etc.).

Another important indicator is mental health literacy. Mental health literacy is the ability to obtain, read, understand, and use health care information to make appropriate decisions, seek help, and follow treatment instructions (Jorm et al., 1997). In the field of health literacy, the connection between mental health literacy and help-seeking behaviors is widely confirmed (Wei et al., 2015). It is therefore also recommended that these be anchored in school education in order to reduce barriers to seeking help and counteract the help-negation effect (not utilizing needed help) (Rickwood et al., 2005). However, further studies are needed to examine the course of people’s help-seeking behaviors over time or against the background of changing conditions (Wei et al., 2015).

In this study, we measure mental health literacy through young people’s help-seeking behavior and their current mental health literacy by asking different questions on their knowledge about mental health. We also include stress in the mental health literacy definition, since stress and mental disorders caused by stress are very common among emerging adults (Bayram and Bilgel, 2008). Perceived stress is often related to a low quality of life (Marshall et al., 2008).

Finally, we measure mental health through a self-report inventory that rates physical and psychological symptoms and a self-rating questionnaire that assesses impairment due to depressive symptoms.

This paper describes the study protocol for a novel intervention that we developed with the aim to complement the educationally oriented support provided in vocational integration programs with a psychotherapeutic service. We also focus on the feasibility of a clinical trial with emerging adults in vocational integration programs. The model that we developed simplifies early recognition and treatment of mental distress in emerging adults. By integrating a psychotherapist in the vocational integration program, we facilitate the emerging adults’ access to health care professionals. In the intervention that we designed, a psychotherapist holds a weekly group meeting with the participants, so that they become familiar with the psychotherapist and have the opportunity to build a trustful relationship. The goal of the group meetings is to provide and discuss psychoeducational information, to foster active confrontation with one’s own mental health problems, and to teach stress reduction techniques, such as mindfulness-based stress reduction (MBSR) (Kabat-Zinn, 2009).

To measure the outcome of the intervention, we chose the following indicators: work ability, mental health literacy, and mental health.

### Objectives

#### Primary Research Question and Hypotheses

The main objective of this study is to investigate if the intervention—the integration of a psychotherapist in a vocational integration program—facilitates access to the labor market for mentally burdened emerging adults by increasing their work ability and mental health literacy and by improving their mental health.

##### Main Hypothesis

Work ability increases significantly from *t*_0_ to *t*_2_.

##### Secondary Hypotheses

Mental health literacy increases significantly from *t*_0_ to *t*_2_. Mental health increases from *t*_0_ to *t*_2_.

Another objective of this study is to determine the feasibility of the intervention and whether it is possible to conduct a clinical trial with unemployed emerging adults who are facing numerous challenges in their transition to adulthood. This study aims to identify implementation difficulties and establish procedures for a potential large-scale trial, including the recruitment of a control group receiving treatment as usual.

The aims are to evaluate the feasibility of the recruitment process and study uptake. Furthermore, we analyze the acceptability and credibility of the therapeutic concept in a non-clinical setting among professionals in vocational training and the emerging adults in these programs.

## Materials and Methods

### Study Design

This is a single-center naturalistic study involving a repeated measure design with three assessments (pre-intervention, post-intervention, and 6-month follow-up). Six month is a common time period for follow up assessments. It allows to collect meaningful data and the risk for ‘lost to follow up’ is reduced compared to longer time periods.

### Recruitment

Participants entering the vocational integration program aged 16–29 years will be recruited for the study by their vocational integration program coach and provided with initial information on the ongoing study. If they agree to participate in the study, the coach will arrange a meeting between the participant and the researcher. Prior to enrollment, all participants are required to read the study information and sign a consent form. Participants will then complete a pre-intervention questionnaire; completion of the questionnaires will take 20–40 min. The researcher, who is a trained psychologist, conducts the clinical interview (duration 45–90 min). The goal of the interview is to obtain a detailed assessment of the participants’ psychopathology; the data will be used only in the descriptive analysis. This first assessment takes place in the first few weeks after the participants have entered the vocational integration program. Alongside the vocational integration program, the participants then take part in the intervention designed for this study.

When the participants leave the vocational integration program and therefore end the intervention, they will complete a post-intervention questionnaire. To ensure data collection, this second assessment will be arranged by the program coach or the researcher. For the 6-month follow-up, the researcher will be responsible for setting up an appointment with the participant. This study was approved by the Ethics Committee of the Canton of Zurich (reference number KEK-ZH-Nr.2017-00936) on September 12, 2017, and is registered with ClinicalTrials.gov (NCT03328286). Duration of the study is 18 months.

#### Participants

To be eligible for the study, participants must be aged 16–29, must take part in the vocational training program, and must be able to understand and complete the questionnaires and understand the clinical interview and the program content in German. Although emerging adulthood is defined as the period from age 18 to 29 (Arnett, 2014), we decided to include 16- and 17-year-olds, as we wanted to avoid excluding a minority and therefore jeopardizing acceptance of the intervention. We foresee that only few participants will not have reached legal age when entering the program.

Coaches in the vocational integration program will verify eligibility.

### Description of the Intervention

The intervention consists in placing a psychotherapist in an already existing vocational integration program. The therapist will hold group sessions once a week lasting 90 min and use well-established group psychotherapy techniques, such as cohesion (Yalom, 1995), empathy (Rogers, 1980), and therapeutic alliance (Bordin, 1979).

The group sessions will provide a social space to discuss challenges, problems, and burdening issues. The group meetings will use elements of Positive Peer Culture programs (Brendtro et al., 2007). The emerging adults can discuss their problems, seek solutions, and help others solve their problems. The issues are prioritized by the group. The aim is to develop a peer culture in which the participants support each other and take responsibility for each other, so that they can experience appreciation and self-efficacy. In the group sessions, the participants can try out new roles and strengthen their self- and external perception and be supported in their identity development. Depending on the psychological distress of the participants, psychology education topics can also be discussed. The psychotherapist can further develop and adapt the contents of the group sessions to the group. The participants have a high degree of co-determination regarding choice of the topics and contents worked on in the group.

Before and after the group psychotherapy, the therapist is on site to support the participants in case of any emergencies (e.g., suicidal thoughts) triggered by the group psychotherapy. The remaining time that the therapist is present in the vocational integration program can be used for individual consultations. We assume that individual consultations will be rare at the beginning and then increase over time. The therapist’s task is to provide support for the emerging adults. If psychotherapy is indicated, the therapist in our study will help the emerging adult to find a therapist outside the vocational integration program. The therapist in the study will provide support during this transition and ensure that a new therapeutic alliance can be built with the therapist outside the program. Since a therapeutic alliance cannot be forced, if the transition cannot be completed successfully, the therapist will look for new solution with the study participant. Study participants can leave the group psychotherapy and the study at any time with no further explanation required and still continue to take part in the vocational integration program.

### Measures

To evaluate the effectiveness of the intervention, the participants will complete the outcome measures described in the following—most of them self-report scales—at pre-intervention, post-intervention, and 6-month follow-up assessment. Table 1 shows the questionnaires as well as their measurement time point within the study. Before data analysis, the questionnaires will be checked for reliability. If reliability values provide unsatisfactory results, we will perform an analysis of the single items.

**Table 1:** Diagnostic instruments and assessment schedule.

		*t*_0_	*t*_1_	*t*_2_
Mental health	Munich-Composite International Diagnostic Interview (M-CIDI)	x		
	*Allgemeine Depressions-Skala* (ADS)	x	x	x
	Brief Symptom Inventory (BSI)	x	x	x
Mental health literacy	Perceived Stress Questionnaire (PSQ)	x	x	x
	Mental Health Literacy Scale (MHLS)	x	x	x
	General Help-Seeking Questionnaire (GHSQ)	x	x	x
Work ability	Work Ability Index (WAI)	x	x	x
	*Allgemeine Selbstwirksamkeitserwartung* (SWE)	x	x	x
	Rosenberg Self-Esteem Scale (RSES)	x	x	x

#### Munich-Composite International Diagnostic Interview

For the clinical interview, we chose the M-CIDI (Wittchen et al., 1998), which is the German version of the DIA-X developed by the World Health Organization. It is a module-based and very flexible diagnostic tool that can support the user reliably and efficiently in diagnosing mental disorders according to the ICD-10 and the DSM-IV. However, no personality disorders are detected. The interview is fully structured and can be completed by a patient with help of clinicians or trained non-clinical personnel in about 45–90 min.

#### Allgemeine Depressions-Skala

The ADS (Hautzinger et al., 2012) is the German version of the Center for Epidemiological Studies Depression Scale (CES-D). It is a 20-item self-rating questionnaire used to assess impairment due to depressive symptoms over the past week. It measures emotional, motivational, cognitive, somatic, and also motor and interactional symptoms. Each item asks how often a symptom occurred in the past week; response options range from “not at all” to “a lot.” There are cut-off values and norms that can be used for a categorical or diagnostic categorization. The ADS has good internal consistency with a Cronbach’s alpha of 0.85 (Meyer and Hautzinger, 2001).

#### Brief Symptom Inventory

Symptom severity will be measured using the German translation of the BSI (Franke and Derogatis, 2000), a 53-item self-report inventory that rates physical and psychological symptoms in the past week. It is the brief form of the Derogatis Symptom Check-List-90 Revised (Derogatis and Unger, 2010). The nine subscales of the BSI assess the following symptom dimensions: somatization, obsessive-compulsive, interpersonal sensitivity, depression, anxiety, hostility, phobic anxiety, paranoid ideation, and psychoticism. Respondents rate each item on a 5-point Likert scale ranging from 0 (“not at all”) to 4 (“extremely”). Alpha coefficients for the nine primary BSI symptom dimensions have revealed satisfying degrees of internal consistency ranging from 0.70 to 0.89, with an *α* = 0.96 for the Global Severity Index (GSI). Convergent validity has been established (Geisheim et al., 2002), with the BSI showing high intercorrelations with established clinical ratings scales.

#### Perceived Stress Questionnaire

The PSQ consists of 20 items that describe stress-related experiences (Fliege et al., 2001). Respondents rate the frequency of occurrence of the described experiences on a 4-point scale: almost never, sometimes, often, and usually, coded from 1 (“almost never”) to 4 (“usually”). A PSQ index is computed using the formula: raw score–30/90, yielding a score between 0 (lowest level of perceived stress) and 1 (highest level of perceived stress). Four subscales with five items each can be derived (worries, tension, joy, demands), with internal consistency values of 0.80–0.86 (Cronbach’s alpha).

#### Mental Health Literacy Scale

The MHLS is a 35-item measure of mental health literacy (O’Connor and Casey, 2015). The measure assesses ability to recognize disorders, knowledge of how to seek mental health information, knowledge of risk factors and causes, knowledge of self-treatments, knowledge of professional treatments available, and help-seeking behavior. The MHLS has a minimum score of 35 and a maximum score of 160; higher scores indicate greater mental health literacy. The MHLS has been shown to have good internal consistency, with a Cronbach’s alpha of 0.87 and test-retest reliability of *r* = 0.79 (*p* < 0.001) (O’Connor and Casey, 2015). For this study, the questionnaire was translated into German, and items 1 through 15 were left out, being considered too complicated to be understood by the target population.

#### The General Help-Seeking Questionnaire

The GHSQ asks respondents to indicate their level of intention to seek help from a number of individuals (e.g., intimate partner, friend, mental health care professional, religious leaders) on a 7-point scale ranging from 1 (“extremely unlikely”) to 7 (“extremely likely”) (Wilson et al., 2005). A higher score indicates a higher intention to seek help for mental health problems. The GHSQ has been shown to be significantly correlated to seeking access to counseling (*rs*(218) = 0.17, *p* < 0.05). Additionally, the GHSQ has been shown to have good test-retest reliability (*r* = 0.92) (Wilson et al., 2005).

#### Work Ability Index

The WAI measures perceived work ability by a questionnaire-based index (Tuomi et al., 2001). The index is determined based on answers to the following seven items: (1) subjective estimation of current work ability compared with lifetime best; (2) subjective work ability in relation to both physical and mental demands of the job; (3) number of diagnosed diseases; (4) subjective estimation of work impairment due to diseases; (5) sickness absenteeism during the past year; (6) own prognosis of work ability 2 years from now; and (7) psychological resources. The final index score ranges from 7 to 49 and is divided into four work ability ratings: poor, moderate, good, and excellent. In their review article, Radkiewicz et al. (2005) report internal consistencies between 0.54 (Slovakia) and 0.79 (Finland) as part of the NEXT study, with over 38,000 study participants in 10 European countries. This is in line with the results of the German version of Martus et al. (2010), where Cronbach’s alphas ranged from 0.58 to 0.77.

#### Rosenberg Self-Esteem Scale

Self-esteem will be assessed using the German version of the 10-item Rosenberg Self-Esteem Scale (Ferring and Filipp, 1996). The RSES is well validated and is the most commonly used measure of self-esteem. Several studies with different samples have provided evidence of its reliability and stability (Martín-Albo et al., 2007; Mimura and Griffiths, 2007; Sinclair et al., 2010). Respondents rate the items on a 4-point Likert scale ranging from 0 (“strongly disagree”) to 3 (“strongly agree”). Satisfactory characteristic values were obtained with regard to both internal consistency and split-half reliability. The estimation of internal consistency varies between the samples, with values in the range of 0.81–0.87; reliability of the split-half calculation according to Guttman’s and Spearman’s rules is between 0.82 and 0.84, so that the scale can be described as sufficiently reliable (Ferring and Filipp, 1996).

#### General Self-Efficacy

The GSE is a self-report measure of self-efficacy. The 10-item scale was reduced in 1981 from the original 20-item scale developed by Jerusalem and Schwarzer (1992) and has since been adapted for 28 languages. The response format for the 10 items is four options on a Likert scale ranging from “not at all true” to “exactly true.” Responses to the 10 items are summed to obtain a GSE total score that ranges from 10 to 40. Numerous studies have produced good psychometric parameters for the scale. When comparing 25 nations, the internal consistency for the overall sample was 0.86 and 0.81 for the German sample (Scholz et al., 2002).

#### Power and Sample Size Calculation

Forty-three emerging adults will be recruited for this study in the vocational integration program. The number of participants needed is based on an *a priori* power analysis using G*Power software (*F*-test, ANOVA: repeated measures, within factors; 1−β = 0.95, *α* = 0.05, Cohen’s *f* = 0.25), which revealed that the study would require a total sample size of *N* = 43 (Faul et al., 2007). An additional nine participants have been added to this estimated sample size to account for an assumed 20% dropout rate, which increases the required total sample size of *N* = 52. The dropout rate has been chosen according to current standards.

#### Analysis

Besides descriptive data analysis, we will analyze changes in the main outcome measures from pre- to post-intervention and at 6-month follow-up. To analyze the data, we will employ a linear mixed model (LMM). This type of model is particularly useful in settings where repeated measurements are made with the same participants (longitudinal study). Linear mixed models also incorporate both fixed and random effects. Since we are expecting a dropout rate of 20%, a particular advantage of linear mixed models is how they deal with missing values, and we therefore prefer it over more traditional approaches such as repeated measures ANOVA. Participants with partial data sets can be included in the analysis. When testing the hypotheses, the level of significance used will be 5% (*α* = 0.05). Alpha adjustment will be used. The statistical analysis of the data will be done using the software package IBM SPSS Statistics Version 24.

To determine the feasibility of conducting a full clinical trial and implementation of the intervention, we will describe the following aspects of the data: adherence to the group meetings, recruitment feasibility, acceptance of this intervention among professionals and the emerging adults in the vocational integration program. These data will be gathered partially through the psychotherapist (adherence to group meeting) but mostly through interviews at the end of the study. The final report will summarize the data to support a clear decision on whether and how to proceed with a full trial and to inform development of the study procedures to maximize the quality of the trial design.

## Discussion

On-site psychotherapy support in a vocational integration program, as we have planned for in this study, has the potential to address many of the practical barriers to help-seeking behavior in emerging adults. The strengths of the proposed research are threefold: first, we address a research gap by focusing on emerging adults in a vocational integration program. As opposed to students or unemployed emerging adults, this population has not been well studied. Second, several studies have examined rates of use of mental health services by adolescents and emerging adults. However, most have been U.S.-based studies (Kessler et al., 2001). The studies show that the rate of use of services is low among adolescents and emerging adults aged 15–24 and that up to 80% of children and adolescents aged 6–17 do not receive needed mental health services (Kataoka et al., 2002). In Switzerland, the rates of use of mental health services are lower, with 5% of 15- to 25-year-olds reporting using mental health services (Bundesamt für Statistik, 2014). The intervention that we propose in this study is innovative for vocational integration programs. Through the implementation of this low-threshold intervention, we hope to increase the rate of use of mental health services by emerging adults, but we also aim at improving early recognition of mental health disorders and appropriate therapy for emerging adults. Third, the intervention contributes to better integration of emerging adults with mental health disorders in the labor market and thus in society.

Despite these strengths, the use of a single group repeated measure design is a methodological weakness that results in the inability to control for potential confounders. In order to have something similar to a control group, we will compare historical data from previous participants of the vocational integration program with data from the current participants. We will use the data from participants who have attended the program for up to 2 years from the start of our study and do a matching with the current sample. We will not be able to compare many indicators, but we can compare the follow-up solution after leaving the vocational integration program, the length of stay in the program, etc., with the current sample. When making this type of comparison, it must be considered that important external factors such as the current labor market situation can have an impact on the abovementioned indicators.

However, we also face possible problems when recruiting the required sample. Emerging adults with mental health disorders in vocational integration programs are already under a lot of societal pressure. We therefore reckon with a 20% dropout rate. Additionally, they stay in the program from 4 months up to a year; this gives us a limited statistical population. Under these conditions, it would not be feasible to implement the study as a part of a randomized controlled trial. Therefore, the present study aims to test a number of important feasibility questions and to try to overcome the barriers that have been identified. Although this limitation might temper the conclusions regarding the effectiveness of the intervention, the strengths of this study have the potential to bring significant knowledge to research and to public policy.

## Ethics and Dissemination

The study has been approved by the Ethics Committee of the Canton of Zurich (Switzerland). After study completion, the results will be published in international peer-reviewed journals. If they provide valid and reliable information in addition to traditional measures, the findings will be presented at international symposiums with the aim to consider ways to develop new research protocols on emerging adults with mental health disorders in vocational programs.

Plans concerning data sharing do not exist at this time.

## Declarations

### Ethics Approval and Consent to Participate

The Ethics Committee of the Canton of Zurich provided ethics approval for the study (2017-00936). Participants will read an information statement and give informed consent before participating in the study. For participants who have not yet reached legal age, the data protection officer states that young people aged around 14 are assumed to be capable of making judgments with regard to questions that exclusively concern their interests. Therefore, no informed consent from a legal guardian is needed. Any modifications of the trial protocol will be communicated to the Ethics Committee and ClinicalTrials.gov.

## Ethics Statement

This study will be carried out in accordance with the recommendations of Federal Act on Research involving Human Beings, Swiss Ethics Committees on research involving humans. The protocol was approved by the Ethics Committee of the Canton of Zurich. All subjects will give written informed consent in accordance with the Declaration of Helsinki.

## Author Contributions

FS wrote the first and successive drafts of the manuscript. FS and AW contributed to conception and design of the study. AW critically revised the manuscript for intellectual content. All authors read and approved the final manuscript.

### Conflict of Interest Statement

The authors declare that the research was conducted in the absence of any commercial or financial relationships that could be construed as a potential conflict of interest. The Gebert Rüf Foundation, which funded this study, has no corporate funding that could pose a conflict of interest.

## Abbreviations

BSI: Brief Symptom Inventory
CES-D: Center for Epidemiological Studies Depression Scale
GHSQ: General Help-Seeking Questionnaire
GLM: Generalized linear model
M-CIDI: Munich-Composite International Diagnostic Interview
MHLS: Mental Health Literacy Scale
PSQ: Perceived Stress Questionnaire
RSES: Rosenberg Self-Esteem Scale
SWE: Allgemeine Selbstwirksamkeitserwartung
VET: Vocational education and training
WAI: Work Ability Index.
